# Extensive Posterior Wall Isolation on Top of Pulmonary Vein Isolation Guided by Ablation Index in Persistent Atrial Fibrillation Ablation

**DOI:** 10.3390/life13030761

**Published:** 2023-03-11

**Authors:** Francesco Sabatino, Domenico Oriente, Fabrizio Fortunato, Antonio Cascino, Giuliano Ferrara, Giuseppe Sgarito, Sergio Conti

**Affiliations:** 1 Department of Electrophysiology, ARNAS Civico-Di Cristina-Benfratelli, 90127 Palermo, Italy; 2 Department of Cardiology, Azienda Ospedaliera Universitaria Policlinico Paolo Giaccone, 90127 Palermo, Italy

**Keywords:** persistent atrial fibrillation, atrial fibrillation ablation, pulmonary vein isolation, posterior wall isolation, Ablation Index

## Abstract

Background: Durable pulmonary vein isolation (PVI) is recommended for symptomatic paroxysmal atrial fibrillation (AF) treatment, but it has been demonstrated that it may not be enough to treat persistent AF (Pe-AF). Therefore, posterior wall isolation (PWI) is among the strategies adopted on top of PVI to treat Pe-AF patients. However, PWI using contiguous and optimized radiofrequency lesions remains challenging, and few studies have evaluated the impact of the Ablation Index (AI) on the efficacy of PWI. Moreover, previous papers did not evaluate arrhythmia recurrences using continuous monitoring. Methods: This is a prospective, observational, single-center study on patients affected by Pe-AF undergoing treated PVI plus AI-guided PWI. Procedures were performed using the CARTO mapping system, SmartTouch SF ablation catheter, and PentaRay multipolar mapping catheter. The AI settings were 500–550 for the anterior PV aspect and roofline, while the settings were 450–500 for the posterior PV aspect, bottom line, and/or PW lesions. All patients received an implantable loop recorder (ILR). All patients underwent clinical evaluation in the outpatient clinic at 1, 3, 6, 12, 18, and 24 months. A standard 12-lead ECG was performed at each visit, and device data from the ILR were reviewed to assess for arrhythmia recurrence. Results: Between January 2021 and December 2021, forty-one consecutive patients underwent PVI plus PWI guided by AI at our center and were prospectively enrolled in the study. PVI was achieved in all patients, first-pass roofline block was obtained in 82.9% of the patients, and first-pass block of the bottom line was achieved in 36.5% of the patients. In 39% of the patients, PWI was not performed with a “box-only” lesion set, but with scattered lesions across the PW to achieve PWI. AI on the anterior aspect of the left PVs was 528 ± 22, while on the posterior aspect of the left PVs, it was 474 ± 18; on the anterior aspect of the right PVs, it was 532 ± 27, while on the posterior aspect of the right PVs, it was 477 ± 16; on the PW, AI was 468 ± 19. No acute complications occurred at the end of the procedure. After the blanking period, 70.7% of the patients reported no arrhythmia recurrence during the 12-month follow-up period. Conclusions: In patients with Pe-AF undergoing catheter ablation, PWI guided by AI seems to be an effective and feasible strategy in addition to standard PVI.

## 1. Introduction

Pulmonary vein isolation (PVI) is currently recommended for paroxysmal atrial fibrillation (AF) catheter ablation, but persistent AF (Pe-AF) remains a clinical challenge [1,2,3]. In this setting, the guidelines recommend that substrate modification should be considered on top of PVI, but the technical approach is not univocally defined, and various strategies have been proposed [1,2,4,5]. Regardless of which target is chosen, complete and durable elimination of the target should be the goal so as not to leave behind partially ablated tissue that could serve as a site for future arrhythmia recurrence. Among the strategies used to achieve atrial compartmentalization and de-bulking, posterior wall isolation (PWI) allows the reduction in the left atrium (LA) critical mass and suppression of AF triggers and drivers. The LA posterior wall (PW) represents an arrhythmogenic substrate that contributes to the initiation and maintenance of AF. However, the feasibility, safety, and effectiveness of PWI as a Pe-AF ablation strategy are still controversial. Moreover, the impact of contact force (CF) technology on the effectiveness of PWI is not well known, although it is associated with deeper and safer lesions with shorter procedural and fluoroscopy times. The Ablation Index (AI) (Biosense, Webster, Inc. Diamond Bar, CA, USA) is an indicator that combines the CF, radiofrequency (RF) application time, and RF power in a non-linear formula and represents a parameter to evaluate the efficacy and safety of RF lesions. Although AI enables an indirect evaluation of lesion quality and size in real-time, its role in PWI has not yet been widely assessed. Moreover, PWI is usually performed by creating a linear ablation of the LA roof that joins the superior PVs and the LA floor that joins the inferior PVs (“box” lesion set). The endpoint of the “box” lesion set is the bidirectional conduction block of the PW. However, using this technique, reconnection along the lines or recurrence of electrical activity within the PW led to AF relapses and atypical atrial flutter. Therefore, it is conceivable that there are still doubts about the durability of linear lesions in the PW for the promotion of “durable box lesion”. For this reason, point-by-point ablation of the entire PW has been advocated as an alternative strategy to obtain complete and durable PWI. Finally, most previous clinical studies have used data from 24 h Holter ECG monitoring to evaluate the arrhythmic recurrences, and these are usually limited to a 1-year follow-up period. It has been proven that more comprehensive rhythm monitoring, such as insertable cardiac monitors or preexisting devices, may provide more accurate detection of arrhythmic events, even if short or asymptomatic.

## 2. Materials and Methods

### 2.1. Patient Population

Consecutive patients with symptomatic and drug-refractory persistent AF who underwent CF-supported PWI on top of PVI between January 2021 and December 2021 at our center were prospectively recruited. Pe-AF was defined according to the latest guidelines when AF was continuously sustained beyond seven days, including episodes terminated by pharmacological or electrical cardioversion that lasted ≥7 days. We comprehensively reviewed the baseline patient’s clinical characteristics from the medical records, including the mean AF duration. The hospital’s institutional review board approved the study protocol. The study complied with the Declaration of Helsinki, and all patients gave written informed consent before the procedure.

### 2.2. Inclusion Criteria

Patients were included in the study if they were (a) aged 18 years and over; (b) affected by Pe-AF with indication to perform catheter ablation; (c) patients who underwent first-time ablation with the support of the CARTO 3^®^ electroanatomical mapping system (Biosense Webster, Inc., Diamond Bar, CA, USA), including the CF-supported ThermoCool SmartTouch^®^ SF ablation catheter, high-density mapping catheter PentaRay^®^, and Ablation Index^TM^ as marker of the lesion; (d) patients who had undergone ICM (loop recorders) or had a previously implanted device (pacemaker or implantable cardioverter defibrillator); (e) patients who had signed an informed consent form.

### 2.3. Exclusion Criteria

Patients were excluded if they met the following exclusion criteria: (a) they were unwilling or unable to consent; (b) in case of the presence of any contraindications to AF ablation; (c) pregnancy or breastfeeding; (d) comorbidities with life expectancy <1 year; (e) contraindications to oral anticoagulation therapy.

### 2.4. Ablation Procedure

All procedures were performed in the usual sterile manner in an electrophysiology lab under conscious sedation and short-term analgesia using intravenous midazolam hydrochloride and fentanyl citrate. In addition, according to our institutional protocol, we used dexmedetomidine infusion throughout the procedure. The doses of midazolam, fentanyl, and dexmedetomidine were based on our experience and previously published data. All patients underwent pre-procedural transesophageal echocardiography to exclude left atrial and appendage thrombosis. According to our center’s protocol, Class I antiarrhythmic drugs (AADs) were discontinued at least three half-lives before the procedure, and amiodarone was discontinued four weeks before the procedure. All procedures were performed using uninterrupted oral vitamin K anticoagulants with a target international normalized ratio of 2–3 on the day of the procedure, while direct oral anticoagulants were discontinued the day of the procedure and resumed the same day. All procedures were performed under intravenous anticoagulation using intravenous heparin with an initial bolus of 50–100 IU/kg, followed by a 1000 IU/h perfusion. The maintenance dose was titrated to maintain the activated clotting time of ≥300 s and rechecked every 20 min throughout the procedures. Venous access was obtained through the right femoral vein. A 7 F decapolar catheter was inserted into the coronary sinus to guide the transseptal puncture. Double transseptal access to the LA was obtained using a Brockenbrough XS needle and two SL1 8.5 F transseptal sheaths (Abbott Medical, Abbott Park, IL, USA). Three-dimensional reconstruction of the LA and high-density bipolar LA voltage (>1000 points) was performed using the PentaRay mapping catheter. Bipolar LA voltage maps were created in sinus rhythm at the procedure’s beginning and end. PVI was performed with RF energy point-by-point, and the VisiTag settings for all the patients were as follows: the catheter position stability was set at a minimum time of 10 s, with a maximum range of 2 mm, minimum force of 5 g for at least 50% of the time, and lesion tag size of 2 mm. A CF of 5 to 20 g was targeted at each site. Lesions were delivered according to the AI pre-specified settings with an upper-temperature limit of 43 °C, power of 35–40 W, and an infusion rate of 17 mL/min. PVI was performed by aiming for a contiguous circle that enclosed each PV antra, with an interlesion distance of < 6 mm. The AI settings were 500–550 for the anterior aspect of the PVs and the roofline, while the settings were 450–500 for the posterior aspect of the PVs and the bottom line and/or PW lesions. PWI was started by connecting the PV antral lesions with an anterior cranial roof line and a caudal line at the floor level of the LA (“box-lesion”). In addition, our lesion set was not limited to the “box”. Extensive scattered lesions across the PW were delivered to achieve PWI. In accordance with the guidelines [6], ablation of the cavotricuspid isthmus was performed in patients with typical flutter documentation. All procedures were performed under esophageal temperature monitoring (Esotherm Plus, Fiab). RF delivery was interrupted when the endoluminal esophageal temperature increased above 38 °C, which was considered as the cut-off limit. The acute endpoint of the procedure was complete PVI and PWI, as demonstrated by differential blocks using the PentaRay mapping catheter placed sequentially in each of the PVs. After a minimum time of 20 min from the last ablation, ipsilateral PVs were rechecked with the PentaRay to determine if spontaneous PV reconnection had occurred, and these sites were tagged. If overt PV reconnection had not occurred, intravenous adenosine was administered to unmask any sites of dormant conduction. We recorded CF and AI data for PVI and PWI for each procedure. Radiofrequency, fluoroscopy, procedural times, and incidence of procedural and peri-procedural complications (vascular complications, cardiac tamponade, thromboembolism, atrio-esophageal fistulas, phrenic nerve palsy, pulmonary vein stenosis, etc.) data were also collected. At the end of the procedure, after obtaining informed consent, loop-recorder implantation (Reveal LinQ Medtronic, Minneapolis, MN, USA) was performed in all the patients.

### 2.5. Patient Follow-Up

All enrolled patients visited the outpatient clinic after 1, 3, 6, 12, 18, and 24 months. At each visit, a standard 12-lead ECG was performed. Oral anticoagulants were stopped at three months during the follow-up period based on CHA_2_DS_2_-VASc, while AADs were withdrawn at three months (if prescribed at discharge) or continued at the physician’s discretion. In addition, after the 90-day blanking period, data from the ILR, PM, or ICD were remotely collected or collected on-site to evaluate the occurrence of atrial tachycardia (AT), atrial flutter (AFL), and AF episodes. Each follow-up focused on the assessment of atrial arrhythmia-related symptoms and AF burden. Atrial arrhythmia recurrence was defined as any documented episode of atrial tachycardia (AT), atrial flutter (AFL), and AF that lasted longer than 30 s. The AF burden was calculated as the percentage of time in AF between each follow-up visit based on manually adjudicated episodes. Any evidence of arrhythmia observed within three months after ablation was defined as early AF and not considered as arrhythmia recurrence.

### 2.6. Statistical Analysis

This was an observational, prospective, single-center study. The patients’ clinical characteristics are reported as descriptive statistics. Continuous variables are expressed as the mean ± standard deviation. The categorical variables were summarized as percentages. A *p*-value of <0.05 was considered to be statistically significant. Arrhythmia-free (AF/AFL/AT) survival curves were generated by the Kaplan–Meier method for illustrative purposes. All statistical tests were performed using SPSS for Windows 25.0 (SPSS, Chicago, IL, USA).

## 3. Results

**Figure 1:**
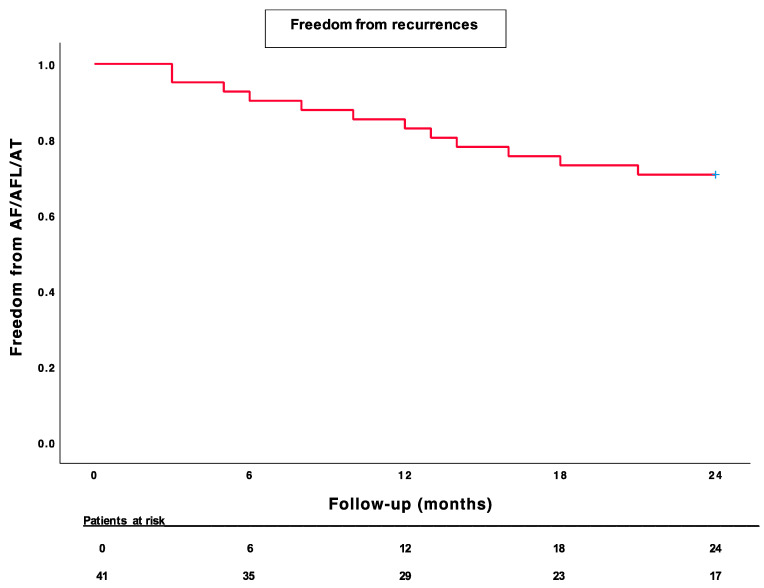
Kaplan–Meier survival analysis.

Forty-one patients underwent PVI plus PWI guided by AI using the CARTO mapping system, SmartTouch SF ablation catheter, and the PentaRay mapping catheter. The baseline clinical characteristics are reported in Table 1. All the included patients had symptomatic (EHRA IIa, IIb and III) Pe-AF with a mean duration of 13.9 ± 2.2 months, and an AF burden of 94.3%. All the patients underwent at least one attempt of electric cardioversion before the procedure. The procedural characteristics are reported in Table 2. The procedure duration was 135.3 ± 18.9 min and the RF time was 31.3 ± 5.4 min. Ablation time on the PW was 7.4 ± 2.2 min. First-pass roofline block was obtained in most patients (*n* = 34, 82.9%), while first-pass block of the bottom line was only achieved in 36.5% of the patients (*n* = 15). Furthermore, PWI was only completed in 39% of patients after the “box-only” lesion, while in the rest of the patients, scattered lesions were necessary to achieve PWI. AI on the anterior aspect of the left PVs was 528 ± 22, while on the posterior aspect of the left PVs, it was 474 ± 18; on the anterior aspect of the right PVs, it was 532 ± 27, while on the posterior aspect of the right PVs, it was 477 ± 16; on the PW, AI was 468 ± 19. No acute complications occurred at the end of the procedure. The average length of hospital stay was 2.2 ± 1.1 days. During the blanking period, the early recurrence of AF occurred in 17.1% of the patients. AADs were discontinued after the blanking period in 75.6% of the patients (*n* = 31/41), while anticoagulation was continued according to the CHA_2_DS_2_-VASc score. After the blanking period, 70.7% of the patients reported no arrhythmia recurrence during the 12-month follow-up period (Figure 1). At the 24 month follow-up appointment, 24.4% of the patients were using AADs. The AF burden significantly decreased from 92% to 24% (*p* < 0.0001).

## 4. Discussion

PVI is the cornerstone in paroxysmal AF treatment, but the optimal strategy in Pe-AF is still debated, and PVI alone seems insufficient to improve patients’ outcomes. The progressive nature of AF requires electrical and structural remodeling, resulting in the development of extra-pulmonary vein arrhythmic substrates [5]. Therefore, the current guidelines recommend that substrate modification should be considered on top of PVI, but the strategy is still debated, not univocally defined, and various approaches have been proposed [6]. Previous studies have demonstrated that PWI is a feasible strategy for catheter ablation of Pe-AF [7,8,9,10,11]. The introduction of AI as a marker of lesion quality to guide PWI in patients undergoing catheter ablation for Pe-AF has been recently advocated. However, the recent CAPLA randomized clinical trial raised doubts about empirical PWI in patients with Pe-AF [12]. Finally, most previous follow-up data are usually obtained via Holter monitoring, event recorders, or transtelephonic monitoring, not continuous rhythm monitoring. To the best of our knowledge, this is the first study performed in patients with Pe-AF undergoing PVI plus extensive PWI guided by AI and combining a rigorous follow-up with continuous rhythm monitoring performed by ILR. This strict follow-up period used in our study has increased our ability to identify arrhythmic recurrences and accurately quantify the AF burden. In our study, 70.7% of the patients reported no arrhythmia recurrence during the 18-month follow-up period. In addition, the AF burden was significantly decreased compared to the patients’ pre-procedural status.

Previous large randomized clinical trials failed to identify a well-defined ablation strategy for Pe-AF patients. The STAR AF II showed no differences in outcomes in patients with Pe-AF between PVI alone, PVI plus linear lesions (roofline and mitral isthmus line), and PVI plus ablation of complex fractionated atrial electrograms. The overall success rate for all three arms in the study was 44%. In addition, the percentage of PV reconnection observed was >80%, which may be related to the technology of ablation catheters available at that time, raising the concern of lesion durability [13]. Of interest, even if the results were disappointing, a sub-study of the STAR AF II showed that the arrhythmic burden was significantly reduced. In fact, by redefining the cut-off of the arrhythmic recurrences documented during the follow-up, the authors showed how the procedural success increased from 44% to 75.1% [14]. The use of CF sensing catheters has been shown to improve both the efficacy and the safety of AF ablation. The TOUCH AF trial was the first randomized, multicenter trial that examined the effect of CF sensing in Pe-AF ablation patients [15]. Despite the minimalist approach adopted in the trial—PVI plus roofline—the authors reported a significantly higher degree of freedom from the arrhythmia recurrence rate than the STAR AF II trial, where no CF catheters were used. In addition to CF sensing information, AI has been developed and proven to improve the effectiveness of AF ablation procedures, making inter-operator differences less heterogeneous [16,17]. Even though the role of AI is well established in PVI, its role in PWI has not been deeply investigated.

The PW of the LA plays a critical role in the initiation and maintenance of AF for several reasons. First, PW contains arrhythmogenic substrates that trigger and maintain AF, due to its common embryological origin with PVs [18]. As the LA develops, the PVs represent the outgrown tissue of the PW. Thus, PVs and PW myocardial sleeves are intertwined, potentially favoring the creation of different and complex circuits. Moreover, specialized conduction tissue with intrinsic pacemaker activity has been found in the myocardial sleeves of the PW [19,20]. Second, there is significant anatomical heterogenicity in the orientation of the myocardial fibers of the PV antra and PW, favoring anisotropic conduction and local reentry. Third, in patients with Pe-AF, PW is an ideal anatomic location for significant atrial remodeling, comprising fibrosis and lymphomononuclear infiltration [21]. Finally, the PW of the LA and PV myocytes have shorter action potential durations [22]. The current strategy that is widely adopted for PWI is derived from surgical ablation. Previous studies reported that patients who underwent a surgical “box” lesion set had greater freedom from AF after one year compared to patients who underwent PVI alone or PVI plus a single connecting lesion [23].

Several studies have demonstrated the feasibility of PWI for catheter ablation of Pe-AF [7,8,9,10,11]. These findings were confirmed in a recent metanalysis of multiple randomized clinical trials, demonstrating the incremental benefit of PWI [24]. However, how to perform isolation of LPW remains a very debatable, controversial issue. There is an intrinsic technical difficulty in providing successful electrical PWI by creating a set of linear lesions due to the complex anatomical architecture of the atrial musculature. Moreover, even if a conduction block along the lines is achieved, the occurrence of gaps over time cannot be ruled out; thus, dormant conduction may take place during the follow-up. Tamborero et al. reported that PWI provided by linear lesions does not improve the clinical outcome of PVI [25]. Nearly 70% of patients in their study demonstrated reconnection of the roof line or recurrence of electrical activity within the PW that led to AF and AFL. Sayuri et al. showed a reconnection of PW in 65% of patients after the second procedure [26].

Recently, the CAPLA randomized clinical trial did not show additional benefits when the empirical PWI was performed in patients with Pe-AF [12]. Nevertheless, there has been some criticism about this study. Patients were included if their duration of Pe-AF was ≤3 years and followed for 1 year. A longer follow-up would have resulted in a better evaluation of the disadvantages of PVI alone after a longer follow-up period. In addition, with the possibility that some patients had relatively early Pe-AF, it is reasonable that they would arguably respond well to PVI as they had paroxysmal AF, and no additional benefit of PWI was evident during follow-up. Furthermore, previous studies have shown a high reconnection rate when PWI was performed using a “box” lesion with 20 to 35 watts. In contrast to CAPLA, the PRECEPT study reported a single-procedure success rate in Pe-AF patients of 80.4% at 15 months, with subsequent improvement in the patients’ quality of life and reduction in hospitalization, which is likely to be due to the different ablation techniques used [27,28].

Finally, most studies have based their follow-up data collection after AF ablation on Holter or transtelephonic monitoring. In the ABACUS study, ILR detected more arrhythmic recurrences than conventional monitoring in AF ablation patients [29]. Similarly, a sub-study of the STAR AF II trial has shown that more rigorous monitoring strategies for detecting AF recurrence after catheter ablation will lower the procedural success rate [30]. In addition, the DISCERN AF study showed that after catheter ablation of AF, the ratio of asymptomatic to symptomatic AF episodes tripled, and the post-ablation state was the strongest predictor of asymptomatic AF [14]. If it is reasonable to state that repeated Holter monitoring following the ablation of Pe-AF may be insufficient to evaluate arrhythmic recurrences, it is also important to perform post-ablation monitoring, maintaining a balance between the benefits, cost, and invasiveness. The additional detection of AF episodes may also be of little clinical relevance, since reductions in AF burdens are typically sufficient to improve patients’ quality of life, hospitalization, and heart function. Another sub-study of STAR AF II showed that by using different cut-off points of arrhythmic episodes during follow-up, the procedural success increased from 44% to 75.1% [31].

## 5. Limitations of the Study

This study has several limitations. First, this was a single-center cohort study. The number of patients included was limited, and we did not include a control group. Larger and randomized data and longer follow-up durations are needed to validate these data. A significant number of patients continued AAD treatment even after the blanking period. The study proves the feasibility of this approach, but we cannot prove any effect of PWI on arrhythmia-free survival, and we cannot give any definitive conclusion on the correlation between PWI and patient outcomes.

## 6. Conclusions

AI is a marker of lesion quality and is characterized by improved lesion formation compared to CF. PWI guided by AI as an adjunctive strategy on top of durable PVI performed during an index ablation of Pe-AF seems to be safe, effective, and reproducible. Larger and randomized studies are needed to confirm the best ablative strategy for Pe-AF patients.

## Figures and Tables

**Table 1:** Baseline clinical characteristics.

	Overall Population (*n* = 41)
Male, *n* (%)	30 (73.1)
Age, mean ± SD	63.6 ± 9.1
Duration of AF, months (mean ± SD)	13.9 ± 2.2
Hypertension, *n* (%)	26 (63.4)
Diabetes, *n* (%)	6 (14.6)
Renal failure, *n* (%)	4 (9.7)
Dyslipidemia, *n* (%)	14 (34.1)
OSAS, *n* (%)	8 (19.5)
COPD, *n* (%)	4 (9.7)
Active smoker, *n* (%)	7 (17.1)
BMI, mean ± SD	28.3 ± 6.8
CHA_2_DS_2_-VASc, mean ± SD	3.1 ± 0.8
HASBLEED score, mean ± SD	1.1 ± 0.8
LA diameter, mm (mean ± SD)	46.3 ± 15.1
LA area, cm^2^ (mean ± SD)	30.4 ± 8.3
LA volume, mL (mean ± SD)	65.3 ± 11.6
Indexed LA volume, mL/m^2^ (mean ± SD)	32.1 ± 5.6
LVEF, mean ± SD	58.4 ± 9.1
Tachycardiomyopathy, *n* (%)	4 (9.7)
EHRA class IIa, *n* (%)	10 (24.3)
EHRA class IIb, *n* (%)	23 (56.1)
EHRA class III, *n* (%)	8 (19.6)
ICM, *n* (%)	4 (9.7)
HCM, *n* (%)	1 (2.4)
*Baseline therapy*	
- Beta-blockers, *n* (%)	30 (73.1)
- Class Ic, *n* (%)	5 (12.1)
- Amiodarone, *n* (%)	28 (68.2)
- Sotalol, *n* (%)	9 (21.9)
Dual-chamber PM, *n* (%)	2 (4.8)

Table legend: AF = atrial fibrillation; OSAS = obstructive sleep apnea syndrome; COPD = chronic obstructive pulmonary disease; BMI = body mass index; LA = left atrium; LVEF = left ventricular ejection fraction; ICM = ischemic cardiomyopathy; HCM = hypertrophic cardiomyopathy; PM = pacemaker.

**Table 2:** Procedural characteristics.

	Overall Population (*n* = 41)
Pre-procedural TEE, *n* (%)	41 (100)
Procedural duration, min (mean ± SD)	135.3 ± 18.9
ICE, *n* (%)	8 (19.5)
US-guided femoral puncture, *n* (%)	6 (14.6)
Double transeptal puncture, *n* (%)	38 (92.6)
LPV common ostia, *n* (%)	6 (14.6)
RPV common ostia, *n* (%)	1 (2.4)
Intermediate/accessory PVs, *n* (%)	1 (2.4)
Adenosine, *n* (%)	41 (100)
*PVI*	
PVI, *n* (%)	41 (100)
WACA, *n* (%)	5 (12.2)
WACA + carina, *n* (%)	36 (87.8)
First-pass PVI, *n* (%)	39 (95.1)
CF on anterior LPVs, (mean ± SD)	13.1 ± 2.9
CF on posterior LPVs, (mean ± SD)	10.8 ± 2.1
AI on anterior LPVs, (mean ± SD)	528 ± 22
AI on posterior LPVs, (mean ± SD)	474 ± 18
CF on anterior RPVs, (mean ± SD)	12.7 ± 2.4
CF on posterior RPVs, (mean ± SD)	11.8 ± 2.6
AI on anterior RPVs, (mean ± SD)	532 ± 27
AI on posterior RPVs, (mean ± SD)	477 ± 16
PV acute reconnection, *n* (%)	2 (4.8)
*PWI*	
RF time on PW, (mean ± SD)	7.4 ± 2.2
First-pass roofline block, *n* (%)	34 (82.9)
First-pass bottom line block, *n* (%)	15 (36.5)
First-pass PWI, *n* (%)	13 (31.7)
Adenosine, *n* (%)	41 (100)
PV acute reconnection, *n* (%)	2 (4.8)
PW acute reconnection, *n* (%)	3 (7.3)
PW area, cm^2^ (mean ± SD)	12.6 ± 4.4
PW voltage at bipolar map, mV (mean ± SD)	2.4 ± 1.1
CF on PW, g (mean ± SD)	12.9 ± 2.3
AI on PW, (mean ± SD)	468 ± 19
Roofline length, mm (mean ± SD)	32.8 ± 3.5
Bottom line length, mm (mean ± SD)	31 ± 5.3

Table legend: TEE = transesophageal echocardiography; ICE = intracardiac echocardiography; US = ultrasound; LPV = left pulmonary vein; RPV = right pulmonary vein; PVI = pulmonary vein isolation; WACA = wide antral circumferential ablation; PW = posterior wall; CF = contact force; AI = Ablation Index.

## Data Availability

Data are available upon reasonable request.
